# EUS-guided glue injection for managing torrential bleeding during gastric neuroendocrine tumor endoscopic submucosal dissection: sealing the storm

**DOI:** 10.1016/j.vgie.2024.07.001

**Published:** 2024-07-23

**Authors:** Radhika Chavan, Zaheer Nabi, Chaiti Gandhi, Maitrey Patel, Rushil Solanki, Milan Jolapara, Sanjay Rajput

**Affiliations:** 1 Ansh Clinic, Ahmedabad, Gujarat, India; 2 AIG Hospital, Hyderabad, Telangana, India

A-24-year-old man with no comorbidities presented with dyspepsia that had been ongoing for 2 months. Findings of an evaluation revealed moderate levels of anemia (hemoglobin:10 g/dL). Gastroscopy showed 2 umbilicated lesions, the largest approximately 1.5 cm to 2 cm in the proximal body and another 8 mm on the lesser curvature of the stomach with no changes of atrophic gastritis. The rapid urease test result for *Helicobacter pylori* was negative. Biopsy samples of the lesions were positive for neuroendocrine tumor (NET) with Ki 67 index <1% to 2%. A serum gastrin level test was performed after the biopsy report, and it was elevated (450 ng/mL). Contrast-enhanced CT of the abdomen showed 2 enhancing lesions, the largest measuring 18 × 12 mm in the proximal body of the stomach on the greater curvature. The ^68^Ga-DOTATOC tracer showed increased uptake in the proximal body lesion only.

The patient was diagnosed to have type II gastric NET without any metastasis. His case was discussed in a multidisciplinary meeting. Because of his refusal to undergo surgery as the result of his young age and also because the NET was <2 cm, endoscopic submucosal dissection (ESD) was planned.^1^ After informed consent was obtained, ESD was attempted with the patient under general anesthesia. Circular markings were made around the lesion using forced coagulation (effect setting 3) with a marking probe (FTRD System; Ovesco Endoscopy AG, Tübingen, Germany) (Fig. 1). Methylene blue mixed with normal saline was injected submucosally using a sclerotherapy needle (Fig. 2), resulting in a good submucosal lift. An incision was made with a DualKnife J (Olympus America, Center Valley, Pa, USA) using the Endo cut I mode (Fig. 3). During the distal extension of the incision, bleeding started (Fig. 4), which became profuse in a span of seconds. The knife was replaced with a hemostatic forceps. However, because of the severe bleeding, the source could not be localized. The gastroscope was withdrawn, and an over-the-scope clip (Ovesco Endoscopy AG) was mounted on the scope and reintroduced into the stomach. The patient’s position during the procedure was changed from the left lateral to supine, semiprone, and reverse Trendelenburg positions to localize the bleeding source. However, despite the change in position, the bleeding source remained submerged in a pool of blood and could not be localized (Fig. 5). Meanwhile, the patient developed hypotension (70/40 mm Hg) and tachycardia (heart rate: 150 beats/min), so further changing of his position was avoided and procedure was continued in the left lateral position. He received intravenous fluids, a vasopressor (noradrenaline), a proton pump inhibitor, and 2 units of packed cell volume.

**Figure 1:**
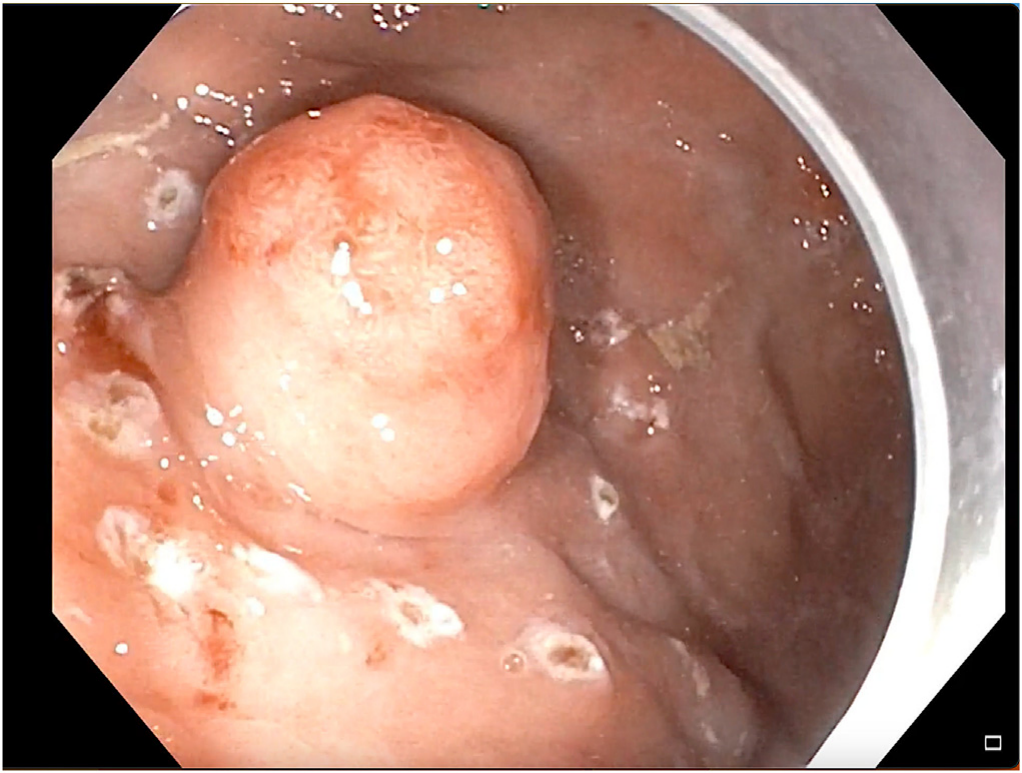
Markings were made in a circular fashion around the gastric neuroendocrine tumor.

**Figure 2:**
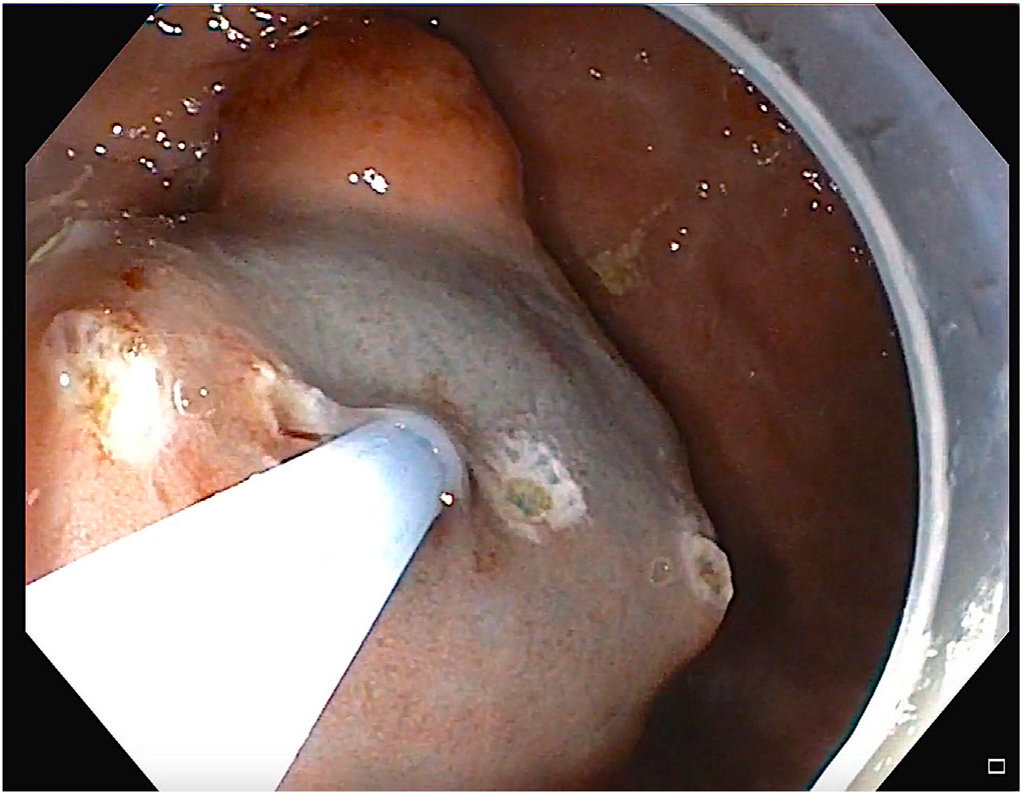
Submucosal injection of methylene mixed with saline was made near the base of the lesion. With this injection, adequate submucosal lift was seen.

**Figure 3:**
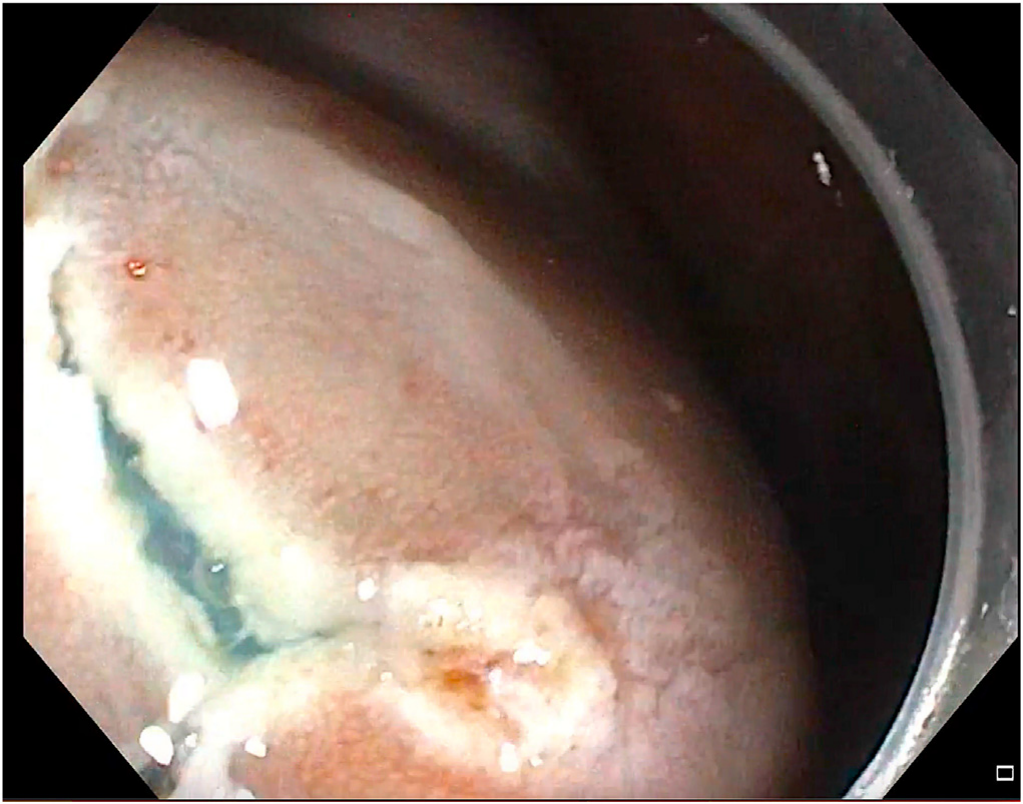
An incision in the mucosa was made by connecting the markings on the lateral side.

**Figure 4:**
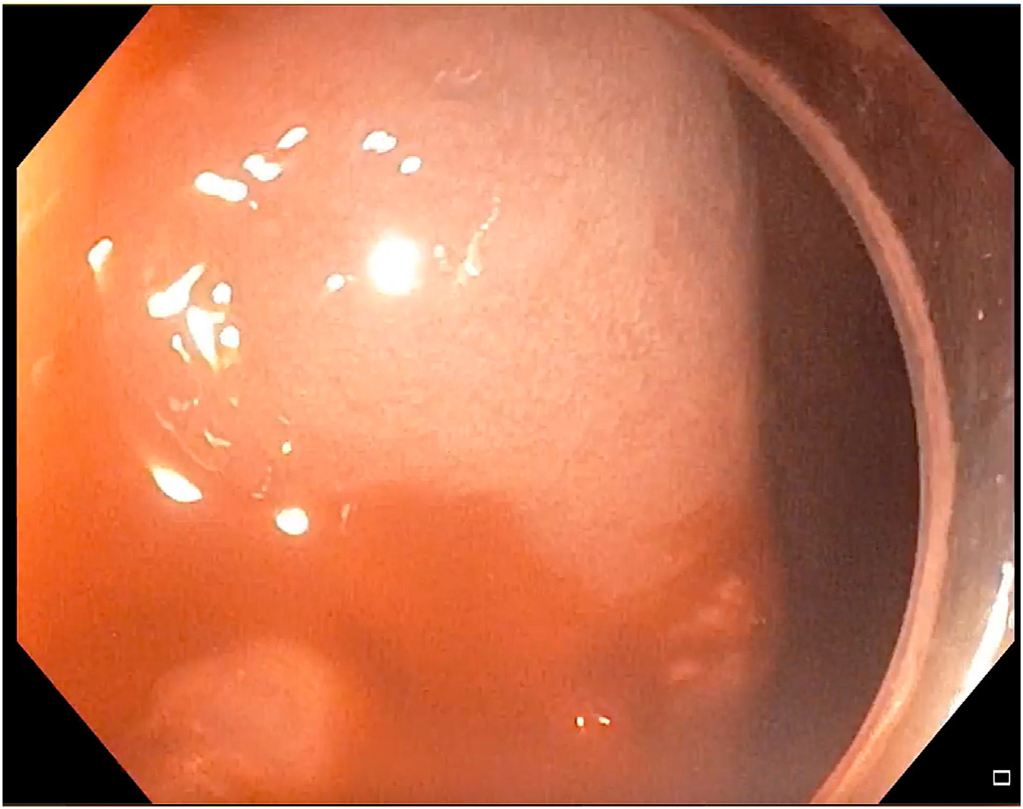
Endoscopic view showing bleeding during the incision in the mucosa.

**Figure 5:**
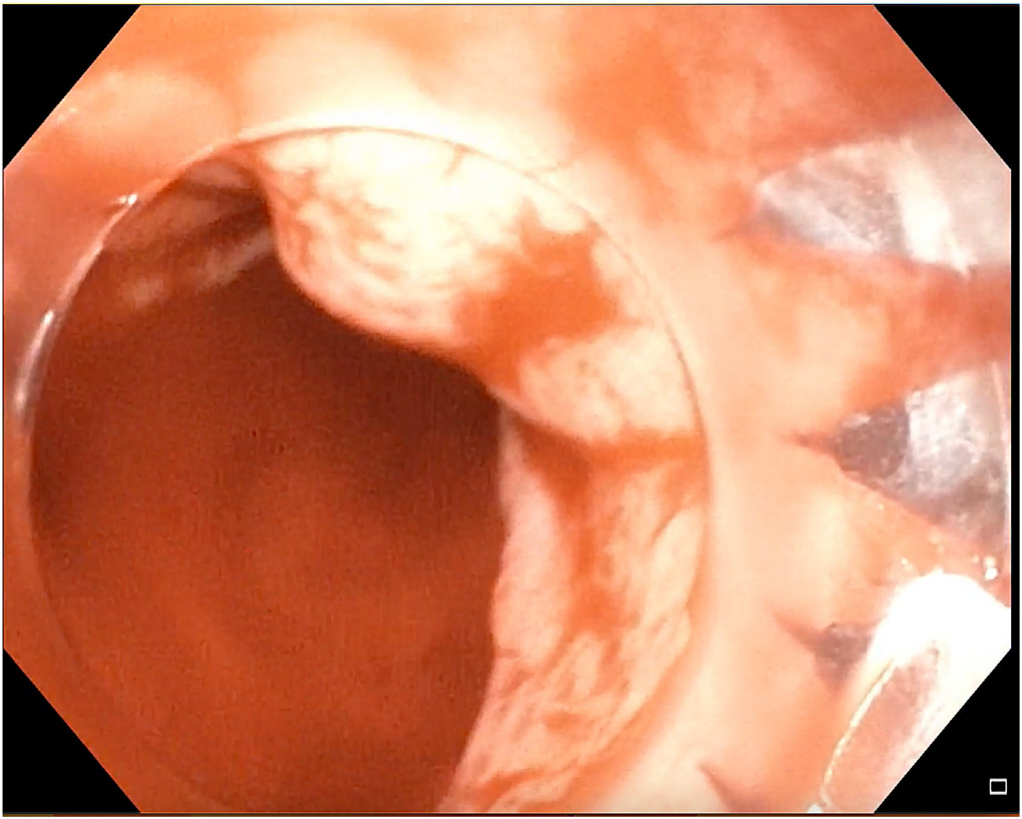
A gastroscope with a mounted over-the-scope clip was introduced into the stomach to control the bleeding; however, the source of bleeding was submerged in a pool of the blood and was difficult to localize.

An interventional radiologist and surgeons were consulted, and arrangements for transfer were made. However, because of the patient’s hemodynamic instability, the risk of death was contemplated. In the meantime, a linear EUS was used to localize the bleeding source. Large hyperechoic clots were seen, and a well-defined hypoechoic lesion was identified in the proximal body of the stomach. On manipulating the echoendoscope, a strong Doppler flow was seen into the gastric lumen from the gastric wall suggestive of active bleeding (Fig. 6). The bleeding source was localized and punctured with a 19-gauge needle, and 1 mL of glue mixed with lipiodol was injected. The glue and lipiodol were mixed as a 1:1 proportion into the 2-mL syringe, and the syringe was shaken well to create a uniform mixture. The preparation of the glue and precautions during EUS-guided glue injections were followed, as previously explained.^2^ After the glue injection, the needle was flushed with 2 mL of distilled water, and the needle was withdrawn into the sheath. After that, the sheath was pushed out of the working channel, and the FNA needle was removed from the scope.

**Figure 6:**
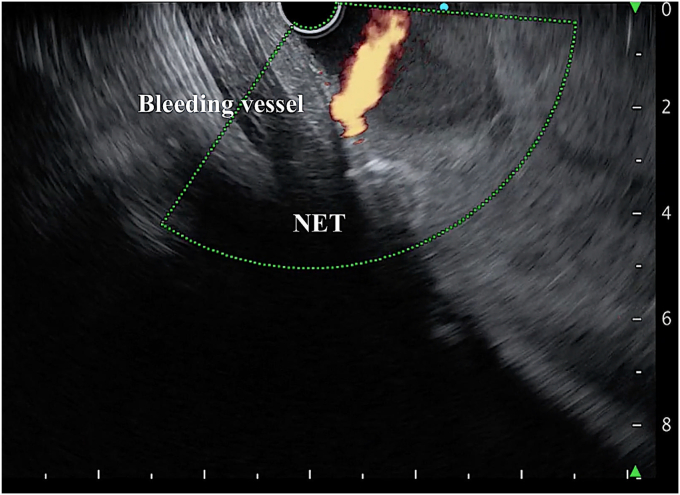
EUS image showing a well-defined NET and adjacent strong Doppler flow from the gastric wall into the lumen suggestive of active bleeding from the shivered submucosal vessel. *NET*, Neuroendocrine tumor.

After the first injection of glue, the bleeding slowed; however, mild oozing was seen on the Doppler flow. Therefore, another 1 mL of glue mixed with lipiodol was injected, and this time needle overshot, injecting glue while the needle was withdrawn (Fig. 7). After the second injection of glue, complete cessation of the Doppler flow was confirmed and the echoendoscope was then replaced with the gastroscope. As the bleeding ceased, the source was localized (Fig. 8). For additional safety, 0.5 mL of glue was injected with the use of the gastroscope (Video 1, available online at www.videogie.org). Further ESD was not attempted because of severe bleeding leading to hemodynamic instability.

**Figure 7:**
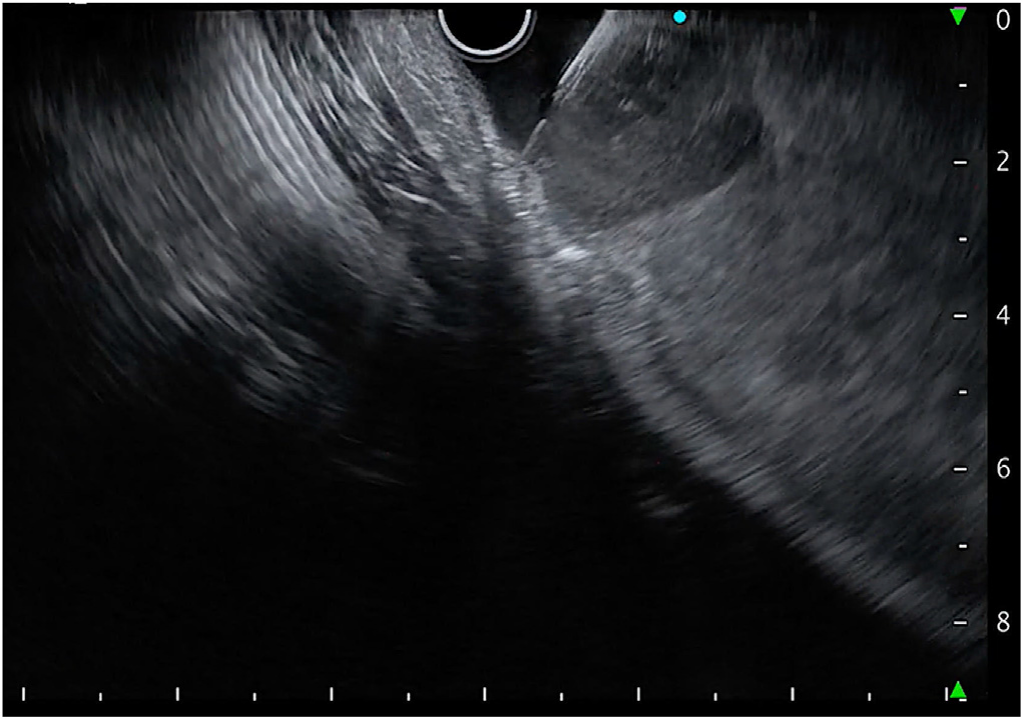
A bleeding vessel was targeted with a 19-gauge needle under EUS guidance, and glue mixed with lipiodol was injected.

**Figure 8:**
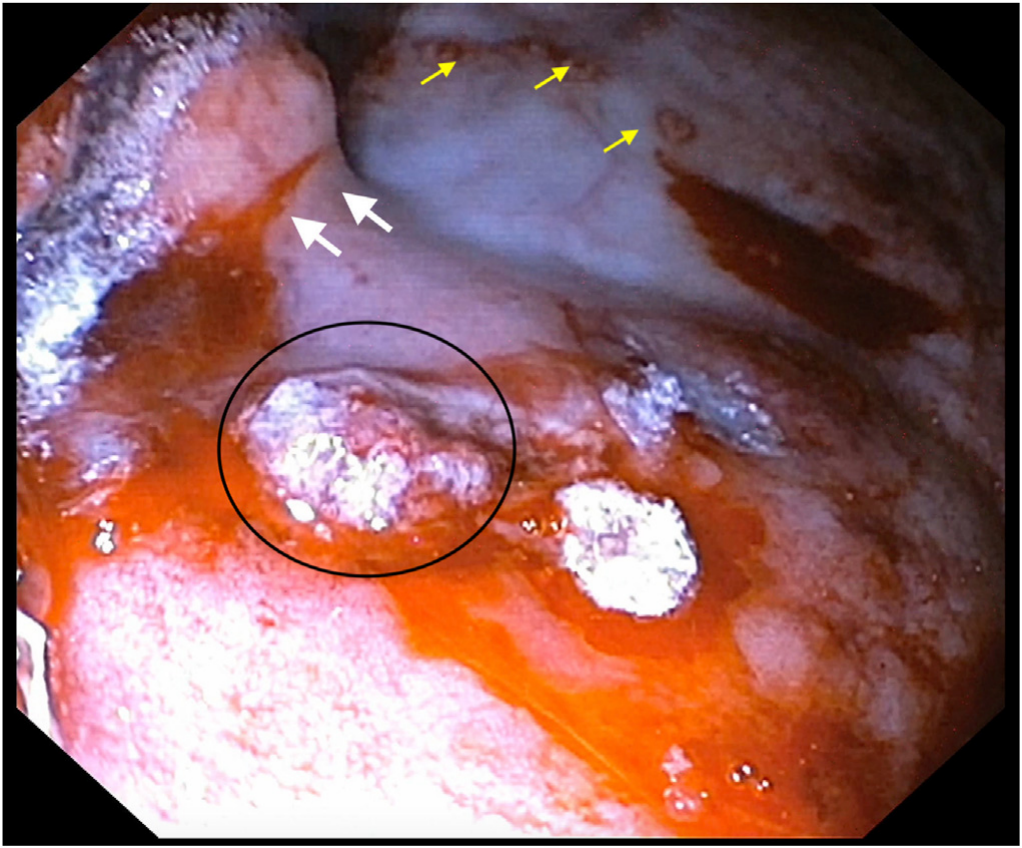
Gastroscopy showed cessation of bleeding, and the bleeding source could be clearly visualized (*black circle*). Markings (*yellow arrows*) and lesion (*white arrows*) also can be seen.

**Figure 9:**
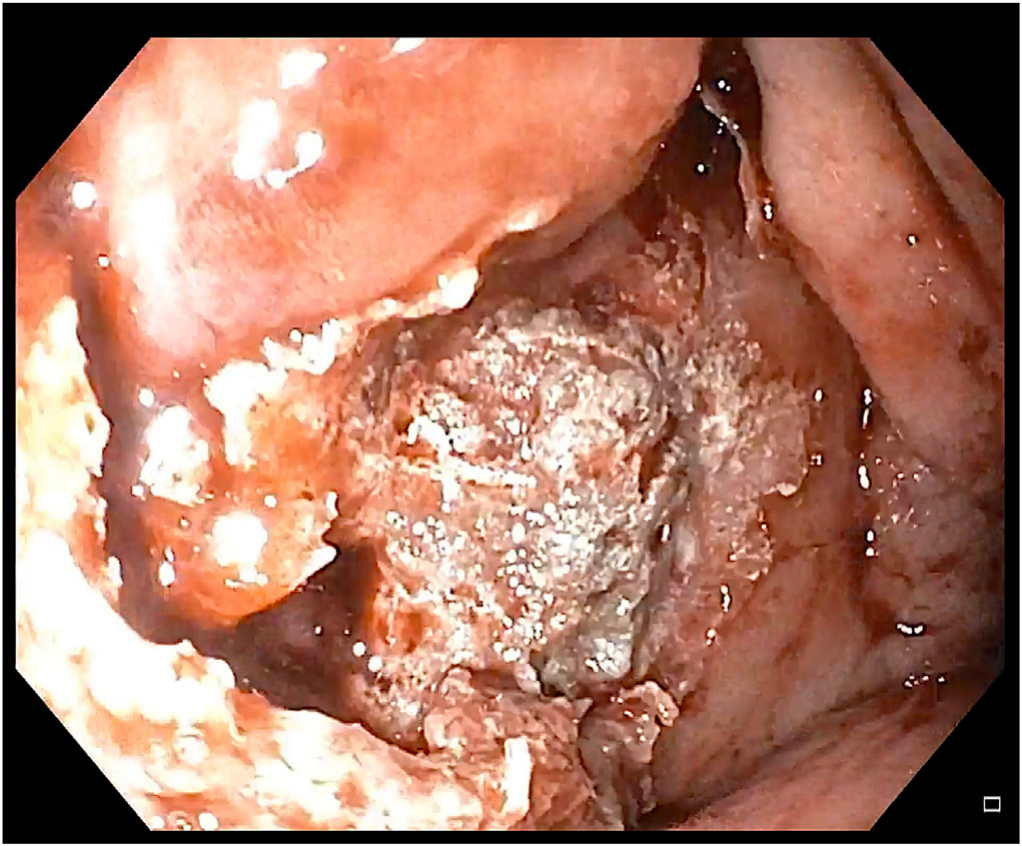
A recheck gastroscopy procedure showed complete cessation of bleeding with glue cast at the site of bleeding source.

During and after the procedure, all supports were continued. A recheck gastroscopy procedure, performed after half an hour, showed no bleeding (Fig. 9). The patient was transferred to the intensive care unit. After transfusion, he developed pulmonary edema, which was resolved with an injection of furosemide. There were no further episodes of bleeding or a decrease in hemoglobin levels noted during the hospitalization. All supports were gradually tapered after the patient was stabilized. He was weaned off the ventilator on day 2, gradually resumed a liquid and then solid diet, and was discharged in stable condition on day 4. Contrast-enhanced CT of the abdomen was evaluated retrospectively for the feeder vessels. Despite the observation of a very faint small gastric arterial branch originating from the splenic artery and coursing near the lesion, it was not possible to definitively confirm it as the feeder vessel of the lesion. The patient has been asymptomatic for 3 months and has not undergone any endoscopic resection or surgery.

Gastric NETs are being detected more often as the result of increased use of gastroscopy for clinical and health care screening purposes. The management of gastric NET includes active surveillance, endoscopic resection, or surgery on the basis of the size, grading, and types of NET.^3^^,^^4^ In this patient, the tumor was grade I with a size <2 cm; therefore, after discussion with the multidisciplinary team, ESD was offered. NETs are vascular tumors; therefore, bleeding during endotherapy is common. However, torrential bleeding during gastric NET ESD is uncommon.^1^^,^^5^ Mild bleeding during ESD can be managed with compression from the distal cap of the endoscope, epinephrine injection, and coagulation with electrosurgical knives; however, major bleeding necessitates the use of hemostatic forceps through the scope or over-the-scope clip. Hemostatic powders (Cook Medical, Winston-Salem, NC, USA) can be an option when bleeding is mild and diffuse. All these techniques are useful if the source of bleeding is localized. In this case, the bleeding source was submerged into the pool of blood and was difficult to localize. EUS is a valuable tool in cases of torrential bleeding in which the source of bleeding cannot be readily visualized. EUS facilitates precise localization of the source of bleeding, even within a pool of blood, and guides therapeutic interventions. Injection of glue should be reserved as a last resort for such bleeding because of its embolization risk. Glue injection into a superficial vessel can cause mucosal ischemia, necrosis, and ulceration. Minimizing the volume of glue can reduce the risk of embolization.^6^

## Disclosure

All authors disclosed no financial relationships.

## Supplementary data

Video 1 Demonstrated is an EUS-guided glue injection for managing torrential bleeding during gastric NET endoscopic submucosal dissection.
